# Viral Impact in Autoimmune Diseases: Expanding the “X Chromosome–Nucleolus Nexus” Hypothesis

**DOI:** 10.3389/fimmu.2017.01657

**Published:** 2017-11-28

**Authors:** Wesley H. Brooks

**Affiliations:** ^1^Department of Chemistry, University of South Florida, Tampa, FL, United States

**Keywords:** autoimmune disease, polyamines, nucleolus, virus, X chromosome

## Abstract

Viruses are suspected of significant roles in autoimmune diseases but the mechanisms are unclear. We get some insight by considering demands a virus places on host cells. Viruses not only require production of their own proteins, RNA and/or DNA, but also production of additional cellular machinery, such as ribosomes, to handle the increased demands. Since the nucleolus is a major site of RNA processing and ribonucleoprotein assembly, nucleoli are targeted by viruses, directly when viral RNA and proteins enter the nucleolus and indirectly when viruses induce increased expression of cellular polyamine genes. Polyamines are at high levels in nucleoli to assist in RNA folding. The size and activity of nucleoli increase directly with increases in polyamines. Nucleolar expansion due to abnormal increases in polyamines could disrupt nearby chromatin, such as the inactive X chromosome, leading to expression of previously sequestered DNA. Sudden expression of a large concentration of Alu elements from the disrupted inactive X can compete with RNA transcripts containing intronic Alu sequences that normally maintain nucleolar structural integrity. Such disruption of nucleolar activity can lead to misfolded RNAs, misassembled ribonucleoprotein complexes, and fragmentation of the nucleolus. Many autoantigens in lupus are, at least transiently, components of the nucleolus. Considering these effects of viruses, the “X chromosome–nucleolus nexus” hypothesis, which proposed disruption of the inactive X by the nucleolus during stress, is now expanded here to propose subsequent disruption of the nucleolus by previously sequestered Alu elements, which can fragment the nucleolus, leading to generation of autoantigens.

## Introduction

Previously, we presented the “X chromosome–nucleolus nexus” hypothesis (1–3). In the hypothesis it was proposed that enlargement of the nucleolus in response to cellular stress could disrupt neighboring chromatin, such as the inactive X chromosome. As a result, sequestered alleles (e.g., polyamine genes on the inactive X), elements (e.g., Alu elements), and viruses could be opened for transcription. This could lead to eventual creation of autoantigens due to overexpression of genes and elements from both the previously active chromatin and the, now, reactivated chromatin. Here is presented new details to the hypothesis, explaining how the disrupted chromatin can lead to subsequent disruption of the nucleolus, even nucleolar fragmentation, which results in ineffective nucleolar functioning, misfolded RNAs, misassembled or incompletely assembled ribonucleoprotein (RNP) complexes, and stabilization of nucleolar components in autoantigenic conformations. Many of the major autoantigens in autoimmune diseases like systemic lupus erythematosus (SLE) are, at least transiently, components of the nucleolus (e.g., splicosome subunits). Among the factors that could cause extraordinary cellular stress, viruses are highly suspected of causing such disruption in autoimmune diseases.

## Viral Involvement in Autoimmune Diseases

Exposomics is the study of all environmental factors which a person may encounter during their lifetime, even including prenatal exposure. These environmental factors in the exposome can include components of the diet, gut microbiota, chemicals, air pollutants, heavy metals, and infectious agents. These factors can cause cellular stress and can have a cumulative effect in cells through accumulation of genetic damage and/or disruption of epigenetic control, especially in genetically predisposed individuals, that establishes the conditions for eventual manifestation and progression of an autoimmune disease. Within the exposome is the infectome which is the collection of pathogens that may contribute to an individual’s onset and progression of an autoimmune disease (4). This can be complicated by the latency of some pathogens and the synergistic effects of multiple pathogens. However, unless one is looking for pathogen antigens, the specific pathogen and its effect on the immune system may be masked by a larger response to more abundant autoantigens some of which the pathogen’s antigens may mimic. In addition, it has been difficult to prove these associations since, in the case of viruses, many viruses can establish latent infections but the autoimmune disease may not manifest itself until several years after the initial infection, thus clouding the true extent of their association. For example, a mononucleosis infection, a.k.a. the “kissing disease,” which involves the Epstein–Barr virus (EBV), increases the risk for subsequent appearance of multiple sclerosis (MS) but manifestation of the MS might not occur until as long as 30 years after the mononucleosis episode (5). Add to this the fact that in the interim the individual has had other infections caused by other pathogens that complicate the situation, potentially triggering the actual autoimmune disease for which an initial EBV infection set the stage. A subsequent infection with another virus could allow activation of latent viruses giving a combined stressful impact on the cell. For example, a primary infection by cytomegalovirus can lead to reactivation of latent EBV which provokes an immune response (6). The induction of one latent virus by another virus shows the potential complexity underlying autoimmune diseases. The general population has had exposure to many of the viruses associated with autoimmune diseases but for most individuals there is no autoimmune disease development, suggesting that genetic susceptibility is also important as well as possible epigenetic and environmental factors. As an example, most adults have had exposure to EBV but few develop an autoimmune disease, suggesting other factors are involved rather than just EBV. A genetic possibility for these differing responses may be based on different HLA types, for example, entry of EBV into a host cell *via* binding of the EBV’s gp42 glycoprotein to human CD21 and lymphocytic antigen type HLA-DR. Other HLA sub-types may have different expression levels or have different affinity for the gp42 and not be as compliant for EBV entry. In addition, the extracellular portion of the EBV BZLF2 protein can suppress antigen presentation by binding HLA-DR delaying detection of the EBV (7).

Table 1 lists many of the viruses that have shown associations with autoimmune diseases. We should note that there is variety in the route of transmission and entry among these viruses: (1) respiratory and oral secretions (saliva, sputum, nasal mucus) (e.g., EBV, parvovirus); (2) gut (e.g., enteroviruses); (3) insect vector transmission (e.g., mosquitos for Zika, West Nile); (4) sexual interactions (e.g., HPV, HIV); and (5) transfusions (e.g., HIV). The tissue types in which viral sequestration occurs may vary, such as EBV behind the blood–brain barrier associated with MS or possible EBV in the synovium associated with RA. We should also note that there are both RNA and DNA viruses listed in Table 1 and most of these viruses can persist in a latent state in the host. Appearance of viral antigens does not necessarily mean that the virus is the cause of the autoimmune disease episode since it may only be the result of stress from an autoimmune disease episode that leads to subsequent activation of a hidden virus.

**Table 1:** Virus and autoimmune disease associations.

Virus	Code	Genome	Family (sub-family)	Genus	Species	Putative autoimmune associations	Reference
Coxsackievirus B1	CV-B1	+ssRNA	*Picornaviridae*	*Enterovirus*	Human enterovirus B	T1D	(8)
Cytomegalovirus (CMV)	CMV (HHV5)	dsDNA	*Herpesviridae* (*betaherpesvirinae*)	*CMV*	Human CMV	AIH, SLE, others^a^	(9–12)
Dengue virus	DENV	+ssRNA	*Flaviviridae*	*Flavivirus*	Dengue virus	Thrombocytopenia	(13)
Echovirus	E	+ssRNA	*Picornaviridae*	*Enterovirus*	Enterovirus B	T1D	(14)
Epstein–Barr virus (EBV)	EBV (HHV-4)	dsDNA	*Herpesviridae* (gamma *herpesvirinae*)	*Lymphocryptovirus*	Human gammaherpesvirus 4	APS, MS, PV, SjS, SLE, RA, others^b^	(11, 15–17)
Hepatitis B virus	HBV	Circular DNA, partially ds	*Hepadnaviridae*	*Orthohepadnavirus*	Hepatitis B virus	AIH, AITD, APS, MS, RA, SLE, RA, T1D	(10, 18)
Hepatitis C virus	HCV	+ssRNA	*Flaviviridae*	*Hepacivirus*	Hepatitis C virus	AIH	(10, 19)
Herpes simplex virus 1	HSV-1	Linear dsDNA	*Herpesviridae* (*alphaherpesvirinae*)	*Simplexvirus*	Herpes simplex virus 1	AIH, ALZ, MS, SLE, others^c^	(10, 11, 20, 21)
Human immunodeficiency virus	HIV	+ssRNA	*Retroviridae* (*orthoret rovirinae*)	*Lentivirus*	Human immunodeficiency virus 1	Impaired CD4 and CD8 cells, autoantibodies	(22, 23)
Human endogenous retroviruses	HERVs	Genomic inserted dsDNA	Human endogenous retroviruses (various groups)	^d^	HERV-Es, HRES-1	AGS, SLE	(24, 25)
Human papilloma virus	HPV	dsDNA	*Papillomaviridae*	*Papillomavirus*	Human papilloma virus	vaccine-associated onset/exacerbation of autoimmune diseases	(26)
Human parvovirus B19	B19	Linear ssDNA	*Parvoviridae*	*Erythroparvovirus*	Human parvovirus B19	APS, RA, SLE, SS	(27, 28)
Human herpes virus 6	HHV-6	dsDNA	*Herpesviridae* (*betaherpesvirinae*)	*Roseolovirus*	Human herpes virus	ACTD, AIH, MS, SjS	(29, 30)
Measles virus	MeV	−ssRNA	*Paramyxoviridae*	*Morbillivirus*	Measles virus	MS	(31)
Varicella zoster virus	VZV (HHV-3)	dsDNA	α*-Herpesvirus*	*Varicellovirus*	Human Herpes 3	MS	(32)
West Nile virus	WNV	+ssRNA	*Flaviviridae*	*Flavivirus*	West Nile virus	MG	(33)
Zika virus	ZIKV	+ssRNA	*Flaviviridae*	*Flavivirus*	Zika Virus	GBS	(34)

*Association may be causative (virus induces autoimmune disease) or result (autoimmune disease facilitates viral expression). Some associations may be due to vaccines (e.g., HPV)*.

*ACTD, autoimmune connective tissue diseases; AGS, Aicardi-Goutiéres syndrome; AIH, autoimmune hepatitis; AITD, autoimmune thyroid diseases including Hashimoto’s and Graves’ diseases; ALZ, Alzheimer’s disease; APS, anti-phospholipid syndrome; GBS, Guilian Barré syndrome; MG, myasthenia gravis; MS, multiple sclerosis; PV, pemphigus vulgaris; RA, rheumatoid arthritis; SjS, Sjögren’s syndrome; SLE, systemic lupus erythematosus; SS, systemic sclerosis; T1D, type 1 diabetes; dsDNA, double-stranded DNA; ssDNA, single-stranded DNA; ssRNA, single-stranded RNA*.

*^a^Others: pharyngitis, lymphadenopathy, and mononucleosis syndrome*.

*^b^Others: giant cell arthritis, Wegner’s granulomatosis, and polyarteritis nodosa*.

*^c^Others: keratitis, herpes esophagitis, and encephalitis*.

*^d^Human endogenous retroviruses (HERVs) are classified *via* differing methods. See Ref. (35). HERV-E (Human endogenous retrovirus, group E); HRES-1 (non-HERV-E human T cell leukemia-related endogenous retrovirus)*.

For most of these associations (Table 1), it remains to be determined if the virus is the causative agent, one of several combined contributing agents, or simply appearing as a result of impaired host cell suppression of the virus. For example, the measles virus is suspected of involvement in MS due to the appearance of antibodies to measles virus antigens in cerebrospinal fluid of MS patients (36). Whether the MS is a direct result of the measles virus or the appearance of measles antigens is a consequence of the MS or is simply coincidental is not known. There may, in fact, be another virus or another environmental agent that has disrupted the suppression of the latent measles virus. As it is, the situation is even more perplexing since the introduction of measles vaccination for the general population has not caused a significant change in the occurrence rate of MS (37). Other viruses with an infrequent association with an autoimmune response can have reemergence in other forms, such as the varicella zoster virus which causes chicken pox and which can reemerge as shingles and is associated with MS (38). And we should bear in mind that human endogenous retroviruses are suspected of involvement in serious diseases, including autoimmune diseases (25, 39).

Among the viruses listed in Table 1, EBV has received the most attention as a virus with links to autoimmune diseases (15, 40). An association with EBV infection has been observed in SLE (41, 42), MS (43), RA, and Sjögren’s syndrome (SjS) (44) and prior EBV infection, as indicated by sero-positivity for EBV antigens, is observed in 94.2% of controls and 99.5% of MS patients (45). In addition, EBV (human gammaherpesvirus 4, HHV-4) is representative of several herpes viruses that have shown associations with autoimmune diseases. Therefore, based on current knowledge, EBV is most useful for describing possible viral involvement in autoimmune diseases in general.

One way in which a viral infection could provoke an autoimmune reaction is by disrupting the host cell’s epigenetic control during a particularly strong cellular stress response to the viral activity leading to expression of previously sequestered gene alleles. Expression from those newly opened sites could lead to imbalance in the protein and RNA products. The female predominance of many autoimmune diseases suggests that the X chromosome and possibly disruption of the inactive X chromosome, a major epigenetic structure in the cell, could be of significance in such a scenario of viral disruption of epigenetic control (1, 3). One point of concern is fragile sites, which are particularly susceptible to viral insertion and DNA breaks. Fragile sites can be hundreds of thousands, even millions of base pairs in length. The X chromosome has four major fragile sites (FRAXA at Xq28; FRAXB at Xp22; FRAXC at Xq22; and FRAXD at Xq27) (1, 46). Reactivation of part or all of the inactive X chromosome could open these fragile sites for expression of hidden viruses in the fragile sites, adding to the cellular stress.

Once a virus becomes active in a cell, one of its prime targets in taking over the cell is the nucleolus. The virus is dependent on the host cell’s machinery, including ribosomes and transfer RNAs (tRNA), in order for viral proteins to be synthesized. And viral RNAs need to be properly folded and assembled into ribonucleoprotein complexes (RNPs). RNA and RNP processing and assembly are major functions of the nucleolus. The virus puts additional demands on the nucleolar functions beyond the host cell’s needs and, since the virus does not code for ribosomes and such, the virus needs to induce increased nucleolar capacity and activity. Localization of viral RNA and proteins to the nucleolus takes advantage of nuclear and nucleolar localization signals (NoLS) and chaperones. In the case of viral RNA transcripts, RNA pol III transcribed RNAs bind SSB/La and SSA/Ro which assist in nucleolar entry and processing along with any required refolding. For viral proteins, nuclear localization signals (NLS), which are sequences of basic amino acids, and NoLS are used but, since these signals are frequently closely positioned, it has been difficult to decipher the NoLS from the more recognizable NLS (47). Some progress has been made in determining NoLS, such as for the adeno-associated virus serotype 2 assembly activating protein (48), and for nucleolar retention signals, such as found in coronavirus nucleocapsid proteins (49).

## The Nucleolus—Structure, Function, and Dynamics

**Figure 1:**
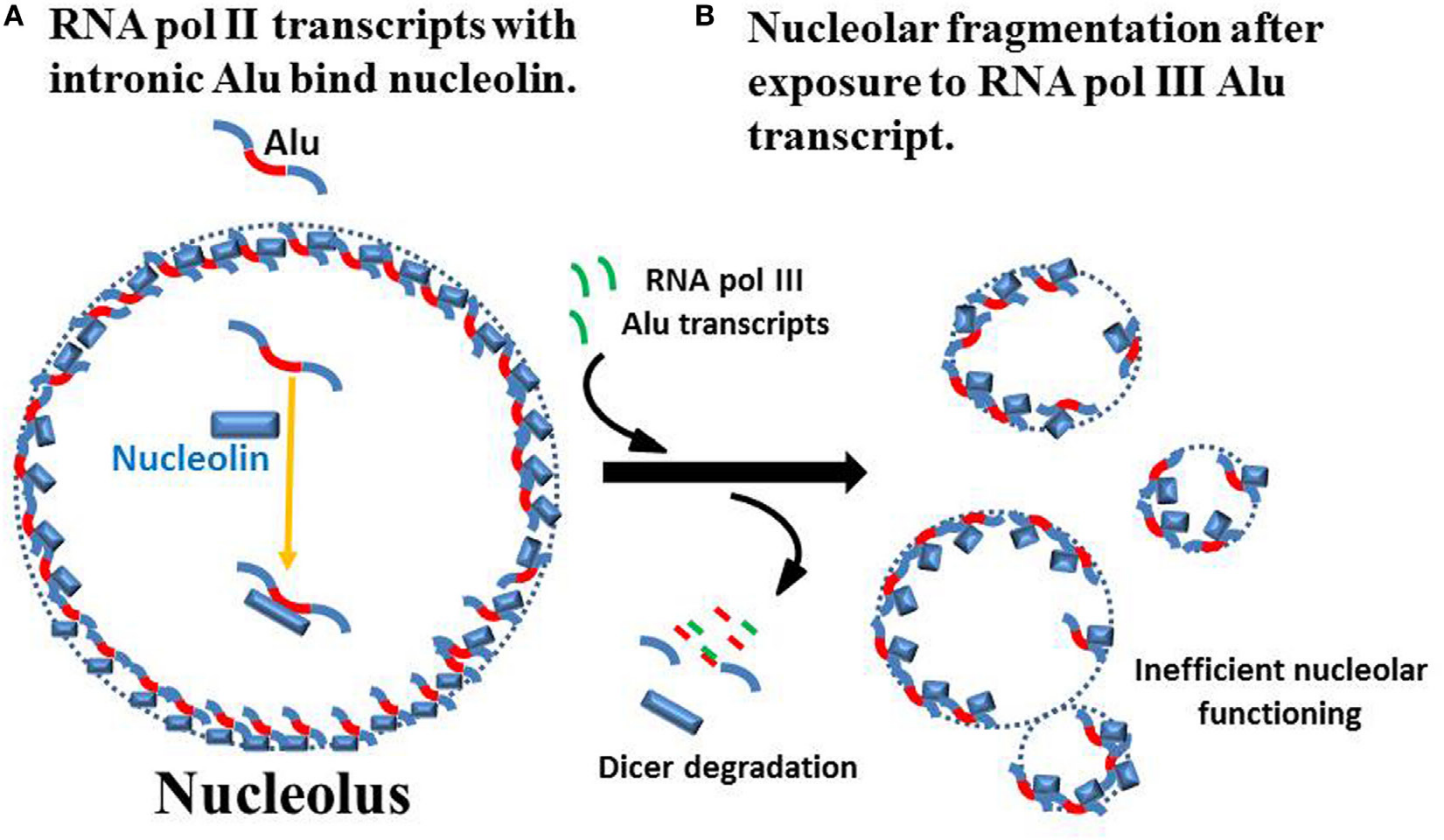
Nucleolar integrity from RNA pol II intronic Alu sequences. **(A)** Caudron-Herger and colleagues reported that the nucleolus has a high content of RNA pol II transcripts with intronic Alu sequences (red) and these transcripts associate with nucleolin to maintain nucleolar integrity (59). (Since the actual localization in the nucleolus and structure of the nucleolin–RNA complexes are not known, they are shown simply as part of the nucleolar perimeter.) **(B)** Addition of RNA fragments from RNA pol III Alu sequences, even as short as 20 bases, leads to loss of nucleolar integrity which Caudron-Herger and colleagues attribute to Dicer degradation of hybridized Alu sequences. This leads to fragmentation of the nucleolus into subunits that are substantially less efficient in nucleolar functions of RNA folding and ribonucleoprotein assembly.

The nucleolus [reviewed in Ref. (50–54)] is a prominent feature in the nucleus, appearing as a vacant area when imaging nuclear DNA content. There is, in fact, some DNA in the nucleolus. As far as active DNA in the nucleolus, nucleoli associate primarily with repetitive copies of ribosomal DNA (rDNA) genes expressing ribosomal RNAs from nucleolar organizing regions (NORs) which are located on the acrocentric autosomes 13, 14, 15, 21, and 22. Other DNA sequences may be involved at the periphery of the nucleolus transiently, such as in repair of breaks in DNA near the rDNA genes. A role in DNA repair in general is emerging for the nucleolus since many proteins involved in DNA repair have associations with the nucleolus (55). Further, centromeric DNA is associated with nucleoli as part of the nucleolar regulation of the cell cycle (56). The nucleolus does not have a membrane defining its structure but nucleoli are typically surrounded by a shell of heterochromatin established in chromosomes containing nucleolar-associated chromatin domains (NADs) (52). This then serves to define the boundaries of the nucleolus. The NADs contain satellite DNA, mostly from centromeric and pericentromeric regions of chromosomes. The NADs also contain gene poor and silent chromatin. In addition, the inactive X chromosome (a.k.a. the Barr body), a heterochromatic body found in most human female cells, is found in close proximity to nucleoli in one-third of cells throughout the cell cycle and 90% of cells in S phase suggesting a putative role for the nucleolus in maintaining X chromosome inactivation (57). The heterochromatin–nucleolar associations are facilitated, in general, by insulator proteins, CTCF (CCCTC-binding factor) and nucleophosmin and additionally, in the case of the inactive X chromosome, by X inactivation specific transcript RNA. EBV latency can be controlled by CTCF bound in the promoter region of the Epstein–Barr virus nuclear antigen 2 (EBNA-2) gene (58). Disruption of chromatin–nucleolar interactions could lead to changes in EBV latency when CTCF interactions with inserted EBV genes are disrupted. In addition, another very important point to keep in mind is that nucleolin bound to RNA polymerase II (RNA pol II) transcripts that contain intronic Alu elements appear to have a critical role in maintaining the integrity of the nucleolus (59). When Caudron-Herger and colleagues added RNA pol III transcribed Alu element sequences, even as short as 20 nucleotides, there was fragmentation of nucleoli into small nucleolar-like units that were very inefficient in carrying out nucleolar functions of RNA and RNP processing and assembly (Figure 1). The authors proposed that the nucleolar fragmentation was attributable to Dicer-facilitated degradation of hybridized RNA pol III Alu sequences with RNA pol II intronic Alu sequences. The work of Caudron-Herger and colleagues demonstrates a close connection between nucleolar integrity and the complexes of nucleolin with RNA pol II transcripts containing intronic Alu sequences. Another possibility for nucleolar disruption by RNA pol III Alu transcripts that Caudron-Herger and colleagues did not mention is possible competition for nucleolin between the RNA pol II intronic Alu sequences and a sudden abundance of RNA pol III Alu transcripts. We believe this could have a major role in generation of autoantigens as we will explain below.

Normally nucleoli contain three discernable sub-regions: the fibrillary centers (FCs), the dense fibrillary centers (DFCs), and the granular components (GCs). The FCs are the sites of rDNA transcription by RNA polymerase I (RNA pol I) to generate the initial ribosomal RNA transcript, the pre-rRNA. Only 50% of the ~400 rDNA repeats in the human diploid genome are transcriptionally active (60). Processing of the pre-rRNA occurs primarily in the DFCs assisted by small nucleolar RNAs (snoRNAs). Assembly of the final ribosomal subunits occurs in the GC, which has a high concentration of proteins needed to complete the RNPs (61). Other RNAs and RNPs processed and assembled in the nucleolus include: the signal recognition particle (SRP) which controls translation and localization of extracellular proteins by transporting them to the endoplasmic reticulum (ER) for eventual extracellular release; tRNAs which require extensive folding; small nuclear ribonucleoprotein complexes involved in splicing of messenger RNAs (mRNAs); and centromere components. Therefore, the nucleolus is involved directly or indirectly in many cellular functions, such as regulation of mitosis; cell-cycle progression; cell proliferation; mRNA processing *via* splicing; translation; protein localization; and various forms of stress response.

The nucleolar proteome contains over 4,500 proteins according to the nucleolar proteome database, NOPdb3.0 (62). About 30% of these proteins are involved in ribosome biogenesis. Since the demands on nucleolar output can change rapidly, the nucleolar proteome is very dynamic. In addition, the size of the nucleolus can change dramatically depending on the needs. Increased nucleolar size correlates directly with increases in polyamine synthesis (63). The polyamines, spermidine and spermine, are involved in many cellular functions but their highest concentrations are found in the nucleoli where the polyamines assist in folding of RNA transcripts and assembly of RNPs. The polyamines have a unique combination of length (spermidine ~11 Å spermine ~14 Å), flexibility (all single bonds) and high cationic charge at physiological pH (spermidine +3; spermine +4) which makes them ideal counter ions to assist in folding the negatively charged RNA transcripts in the nucleolus.

Nucleoli are very dynamic structures in the cell and cell cycle. There can be more than one nucleolus in the nucleus and, combined, they can occupy up to 25% of the nucleus. They can expand rapidly, facilitated by increased polyamines, in response to cellular stress since the cell may need to have more ribosomes and tRNAs to synthesize new proteins to recover from the stress. However, we should remember that the nucleolus surrounds itself with heterochromatin so there is the possibility of displacement or disruption of neighboring heterochromatin due to the nucleolar dynamics (1). With regards to the cell cycle, nucleoli disappear in mitosis and reappear in telophase and early G1 forming around NORs with the rDNA genes and pre-existing rRNA and ribosomal complexes (64). In addition, CDK1 cyclin kinases have a key role in controlling nucleoli during cell cycling, and centromere complexes are generated in the nucleolus giving further importance to nucleoli in cell cycling.

## Viral Impact on the Nucleolus

Once in the host cell, the virus can be sequestered into the host chromatin or it can initiate viral replication. In the case of EBV, the multi-functional Epstein–Barr nuclear antigen 1 (EBNA-1) protein from the EBV genome can assist in the spread and attachment of viral DNA to metaphase chromosomes (65). EBNA-1 can also disrupt the host cell’s USP7-assisted stabilization of p53/TP53, thereby preventing the host cell from entering apoptosis, setting the stage for continual viral replication (65). However, viruses do need host cell machinery produced in the nucleolus, such as ribosomes, to facilitate viral replication. Therefore, the virus will attempt to increase nucleolar activity and turn on viral gene expression. The EBV genome has a snoRNA, called v-snoRNA1, which is found in the nucleolus in infected cells (66). The v-snoRNA1 appears to be involved in activation of the viral DNA polymerase. Another EBV early gene is Epstein–Barr nuclear antigen 2 (EBNA-2) which is a transactivator of viral and host genes. EBNA-2 can associate with RNA polymerase II promoters to induce increased transcription and this includes the *MYC* gene (67, 68). MYC induces increased RNA polymerase III (RNA pol III) activity which creates viral RNA transcripts and many of the RNA transcripts for nucleolar assembled complexes (69). And MYC induces increased transcription by RNA pol I to create rRNA transcripts (70). The MYC interactome consists of approximately 15% of genes throughout the host genome (71, 72). Included among these are genes involved in polyamine synthesis: ornithine decarboxylase (*ODC*); spermidine synthase (*SDS*); and spermine synthase (*SMS*) (73–75). And so the virus induces polyamine synthesis which is directly associated with an increase in the size and activity of the nucleolus (63, 76). The cationic polyamines are ubiquitous and have many important interactions throughout the cell and local extracellular environment (e.g., spermine in neural synapses). The highest levels of polyamines are found in the nucleolus where the polyamines play a critical role in RNA folding by neutralizing anionic charges in the RNA sufficiently for intra-strand RNA–RNA interactions to form. Polyamine availability directly correlates with increased RNA expression and processing (77). And the polyamines can assist in RNP complex assembly. Mostly these are transient interactions of the polyamines but, in some cases, the polyamines remain as part of the final RNA complex, such as in tRNAs (1). Since viral genes need to be expressed and viral RNAs need to be folded and some viral proteins localize to the nucleolus for folding and assembly into the final virion, it is understandable why a virus would want to increase the host cell’s polyamine content to increase nucleolar capacity and activity (78). However, the relation between viral activation and subsequent RNA synthesis is more complex and can vary among viral types. For example, poliovirus inhibits RNA pol I activity by inducing SL1 cleavage and UBF posttranslational modification (79), whereas hepatitis C virus stimulates RNA pol I activity which is involved in transcription of the rDNA genes (60).

Viruses can influence the nucleolar proteome leading to abnormal redistribution of nucleolar components to the nucleus, cytoplasm, and even cell surface (80). For example, viruses induce cell surface exposure of SSB/La which normally facilitates termination of RNA pol III transcription in the nucleus and then, along with SSA/Ro, acts as a chaperone for the RNA transcript as it is processed in the nucleolus (81). Viruses can also disrupt the cell-cycle related kinases in the nucleolus, suppressing normal cell cycling and, thereby, hijacking the nucleolus to focus on viral RNA and protein synthesis and assembly of viral RNP complexes (78). The usual effect of cellular stress, such as viral activity, on the nucleolus is to cause enlargement of the nucleolus but on some occasions the nucleolus can actually decrease in size. Inhibition of RNA pol I by poliovirus, as mentioned above, could be such a situation since a drop in rRNA transcripts would inhibit the major nucleolar function of ribosome synthesis.

## The “X Chromosome–Nucleolus Nexus” Hypothesis: Disruption of the Inactive X

**Figure 2:**
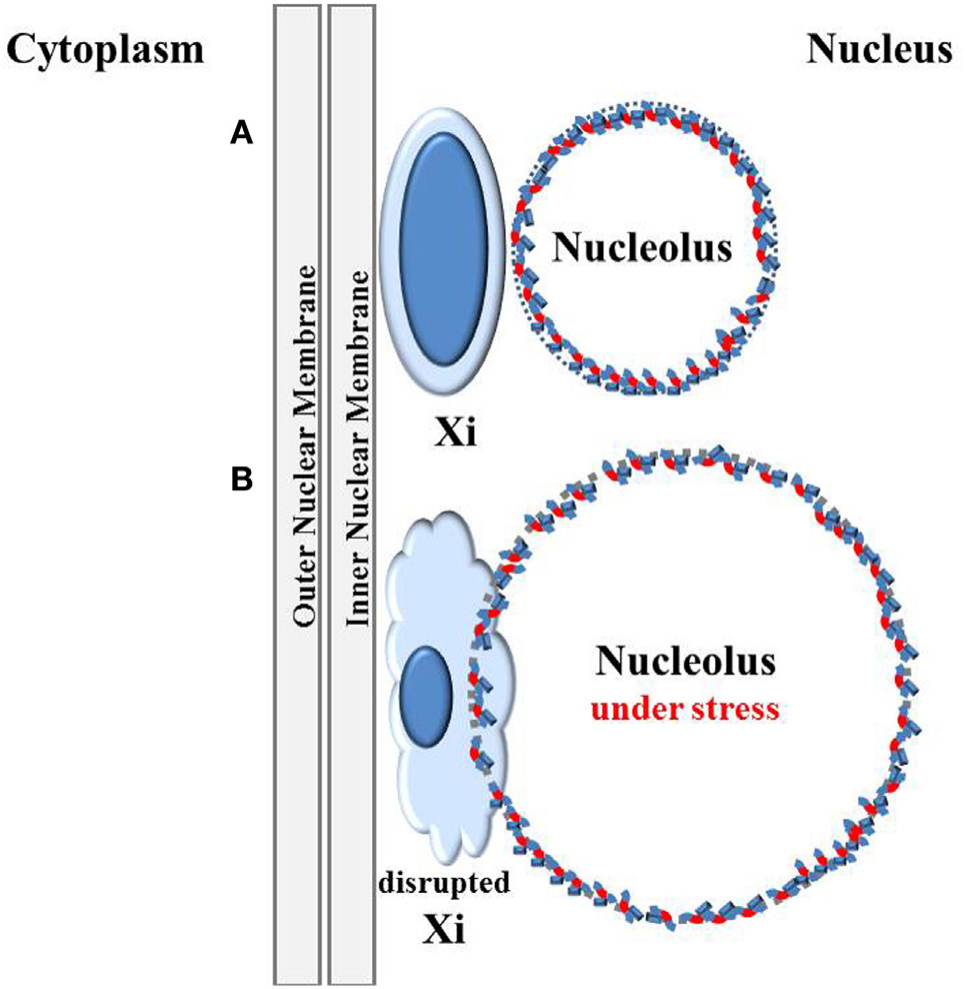
Disruption of the inactive X by the nucleolus under stress. **(A)** The inactive X chromosome (Xi) is typically located at the nuclear periphery next to the nuclear membrane and is associated with a nucleolus in 90% of cells in S phase and one-third of cells throughout the cell cycle except during mitosis when nucleoli disappear (57). This places one of the most inactive structures in the cell, the inactive X, next to one of the most active, multi-functional, and dynamic structures, the nucleolus. **(B)** The nucleolus can rapidly expand during cellular stress, such as viral activation. Trapped between the nucleolus and the nuclear membrane, the Xi could be disrupted by the expanding nucleolus.

The stress that viral activity can put on the nucleolus can lead to extensive enlargement of the nucleolus. This could potentially disrupt the epigenetic silencing in heterochromatin neighboring the nucleolus. The inactive X chromosome, a.k.a. the Barr body, would be especially vulnerable, as we proposed in the original version of the “X chromosome–nucleolus nexus” hypothesis (1), since the Barr body is frequently found in close proximity to a nucleolus (57) and against the nuclear membrane (82), as depicted in Figure 2A. Sandwiched between the nucleolus and the nuclear membrane, the Barr body would not be able to avoid exposure and disruption due to the nucleolin and nucleophosmin that are involved in chromatin remodeling from an expanding nucleolus (83, 84). In addition, exposure of the chromatin to the high content of polyamines in the nucleolus could add to the disruption of chromatin since the cationic polyamines can compete with histones for DNA binding. Moreover, the polyamines have the potential to stabilize alternate DNA conformations, such as Z-DNA, which is targeted by autoantibodies in some cases of SLE and RA. Negative supercoiling stress is stored in nucleosomes as the double-stranded right-hand coiled B-DNA makes a left-handed supercoil over the surface of the nucleosome’s histones. Displacement of the histones during chromatin remodeling could release the negative supercoiling stress, allowing it to flux through the DNA and potentially flipping into left-hand coiled Z-DNA which is also a form of negative supercoiling storage. Z-DNA appears only transiently in chromatin since most DNA is wrapped up as B-DNA in nucleosomes. Z-DNA is not flexible enough to bend around histones so it is excluded from the 145 bp bound to the surface of the histones. Since nucleosomes in human chromatin occur every 200 bp on average, there is normally little opportunity for Z-DNA to form and persist. Exposure to high levels of polyamines from the nucleolus concomitant with disruption of nucleosomes could increase the likelihood of Z-DNA persistence when there is a shift of negative supercoiling storage from nucleosomes to Z-DNA (85). In a similar manner, DNA cruciforms are formed from negative supercoiling stress but their occurrence is also suppressed by positioned nucleosomes. The Alu elements, of which there are more than one million throughout the human genome, contain sequences capable of cruciform formation (1). Since there are approximately 15 million nucleosomes associated with the human genomic DNA, there is ample stored negative supercoiling stress. This potential for rapid dynamic changes in chromatin, including disruption of higher order “stacked” nucleosomes, alternate DNA conformations, displacement of bound proteins, and DNA strand separation, is perhaps under-appreciated aspects of epigenetics and could come into play when the nucleolus encroaches on surrounding chromatin.

**Figure 3:**
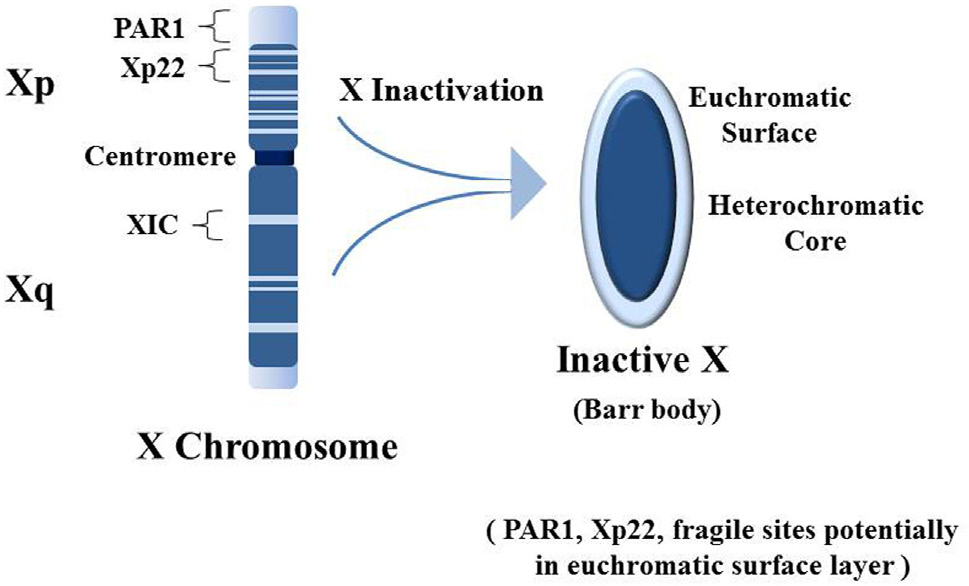
Establishment of the inactive X chromosome (Xi). Early in embryogenesis one of the two X chromosomes in female cells is inactivated by persistent expression of the X inactivation specific transcript RNA (XIST) from the X inactivation center (XIC). XIST RNA does not code for protein but remains in the nucleus and binds contiguous chromatin (i.e., the Xi, a.k.a. the Barr body), recruiting epigenetic silencing effectors (e.g., DNA methyltransferases). Approximately 95% of genes from the long arm (Xq) and 65% of genes from the short arm (Xp) are silenced. Silenced genes shown as dark blue, while genes that escape inactivation are shown as light blue [based on Ref. (89)]. The result is the Barr body which appears as a dense heterochromatic structure near the nuclear membrane. The bulk of the heterochromatic core contains Xq genes with some Xp genes, and the euchromatic-like surface layer has primarily Xp genes that are: actively expressed; potentiated for expression; or silenced but adjacent to expressed genes. Particularly interesting in the Xp is the pseudo-autosomal region 1 (PAR1) which has an abundance of Alu elements (46). In addition, Xp22 contains a “hot” LINE-1 sequence that can code for a fully functional reverse transcriptase. Xp22 also contains a fragile site (FRAXB). Fragile sites are preferential locations for viral insertions. And Xp22 on the Xi contains epigenetically silenced genes for spermine synthase (SMS) and spermidine/spermine N1 acetyltransferase (SAT1). Overexpression of SMS and/or SAT1 that could occur with disruption of epigenetic silencing on the Xi can impact cellular methylation and polyamine types and levels. This could also impact polyamine activity in the nucleoli.

Normally males have only one X chromosome whereas females have two X chromosomes. Most genes on the X are not sex-related so females only need one active X chromosome. Therefore, early in embryogenesis, each female cell inactivates one X chromosome, either the maternally derived or the paternally derived X, and each daughter cell will inherit that inactivation pattern. The process of X chromosome inactivation [reviewed in Ref. (86)], which results in the heterochromatic Barr body, begins from the X inactivation center at Xq13 of the X chromosome’s long arm (Xq) (Figure 3). Approximately 95% of genes on the Xq and 65% of genes on the short arm (Xp) are inactive and form the heterochromatic core of the Barr body (87). The surface of the Barr body would be more characteristic of euchromatin with genes that escape inactivation and some inactive genes that are surrounded by active genes or genes potentiated for activity. Especially interesting are genes on the Xp from Xp22 to the Xp telomere, including the pseudo-autosomal region 1 (PAR1). These would be at the surface of the Barr body and more readily disrupted by an expanding nucleolus under stress. SMS and spermidine/spermine N1 acetyltransferase (SAT1) are involved in polyamine synthesis and recycling, respectively, and normally SMS and SAT1, located at Xp22.1, are inactive on the Barr body (87). However, disruption of the Barr body by enlargement of the nucleolus, as shown in Figure 2B, could lead to reactivation of SMS and SAT1. This would result in a rapid increase in polyamine synthesis and recycling beyond what was already induced by the host cell and the viral activity. There would be an increase in acetylated polyamines by SAT1 that could interfere with RNA folding and, through oxidation, generate putrescine which, in turn, could allosterically increase S-adenosylmethionine (SAM) decarboxylase activation reducing SAM needed for DNA and protein methylation (72). Excess free polyamines can be conjugated to proteins by transglutaminases and acetylated polyamines, and the conjugated polyamines and putrescine can be oxidized to toxic acrolein. There is a close relationship between the intensity of SjS and the appearance of acrolein-conjugated proteins (88). The net effect of expression of SMS and SAT1 from the disrupted Barr body is dysregulation of polyamine levels. Add to this the fact that SAT1 can undergo super induction, meaning there could be a several 100-fold increase in polyamine acetylation. In the nucleolus, there could be an increase in polyamines and now acetylated polyamines that interfere with normal RNA folding and RNP assembly. Once SAT1 becomes active from both X chromosomes from nucleolar disruption of the Barr body, going forward there could be a drop in polyamines during subsequent stress events as super induction of SAT1 acetylates polyamines. It may follow that the nucleolus can no longer expand sufficiently to adapt to new stresses and the nucleolus can no longer work efficiently in proper folding and assembly of RNAs and RNPs, leading to creation of autoantigens. In other words, an initial stress-induced polyamine-driven expansion of the nucleolus could disrupt the Barr body leading to RNA pol III Alu transcript-driven fragmentation of the nucleolus. Subsequently, with reactivation of SAT1 from the Barr body, it may reduce polyamines and reduce the ability of the nucleolus to function normally in folding and assembly of RNAs and RNPs and fail to react effectively to future stressful events since polyamines will be rapidly acetylated as they are synthesized. This scenario could result in ongoing generation of abnormal, potentially autoantigenic RNPs due to the compromised nucleolus that lacks sufficient polyamines.

Another problem that could arise is reverse transcription. Most LINE-1 elements have mutated sufficiently so that they no longer code for functional reverse transcriptases. A few, including one in Xp22, can still produce functional reverse transcriptases but are suppressed by positioned nucleosomes (1). Reverse transcription of Alu elements could be particularly consequential. Alu elements are rich in G–C base pairs; therefore, reverse transcribed Alu DNA would require significant methylation since hypomethylated DNA would be interpreted as exogenous. LINE-1 reverse transcriptases preferentially reverse transcribe LINE-1 RNA at a rate of 1,000× and Alu RNA at a rate of 300× in comparison to other RNAs (90). The cell could quickly become inundated with hypomethylated Alu DNA. Li and Steinman reported a high content of Alu DNA (up to 55%) in the free DNA in sera of lupus patients whereas Alu elements only account for 10.8% of the human genome (91). Those authors suggested that reverse transcription could be a possible cause. We proposed that fully functional LINE-1 elements activated from disruption of the X chromosome could be involved in such reverse transcription.

## Expanding the “X Chromosome–Nucleolus Nexus” Hypothesis: Disruption of the Nucleolus

The earlier version of the “X chromosome–nucleolus nexus” hypothesis suggested that there could be consequences from expression of previously sequestered Alu elements, particularly from the PAR1 of the X chromosome short arm where there is an exceptionally high content of Alu elements (1). We can now add detail to the hypothesis regarding what consequences could arise from RNA pol III expression of these Alu elements.

**Figure 4:**
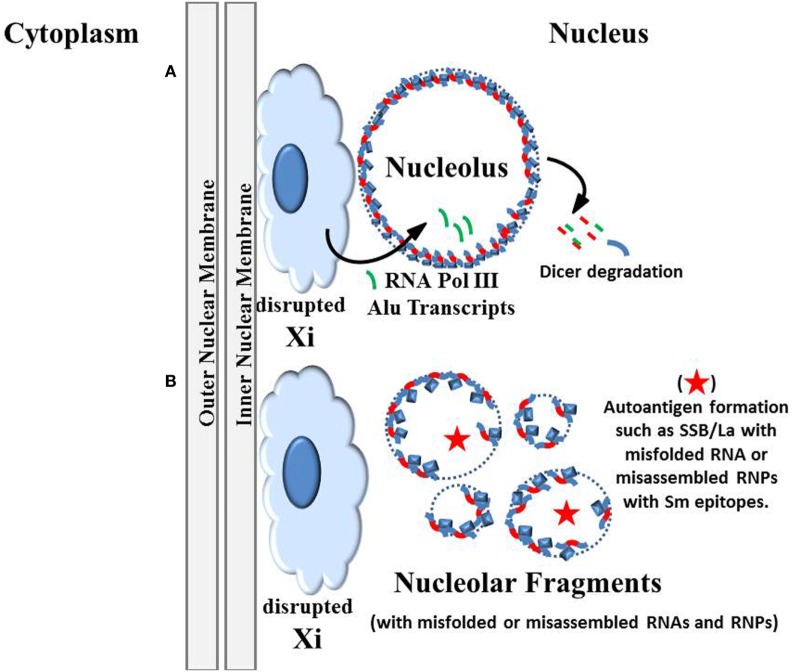
Autoantigens generated by disruption of the nucleolus. **(A)** The original version of the “inactive X chromosome and nucleolus nexus” hypothesis proposed that there is disruption of the inactive X by the nucleolus due to an extraordinary expansion of the nucleolus under stress (Figure 2B). This disruption could open previously sequestered DNA, especially Alu sequences and genes in the short arm of the Xi that are located in the euchromatic-like surface layer of the Xi (1). Now, based on the work by Caudron-Herger and colleagues (59), we can propose additions to the hypothesis, that X-linked Alu transcripts generated by the abundant RNA pol III near the nucleolus can disrupt the nucleolin-RNA pol II intronic Alu complexes, either by Dicer degradation or by direct competition between the RNA pol III Alu transcripts and the intronic Alu sequences. **(B)** The subsequent fragmentation of the nucleolus could result in nucleolar fragments that contain conformationally abnormal autoantigenic structures due to improperly folded RNAs or improperly assembled ribonucleoprotein complexes (RNPs). For example, in some nucleolar fragments there may be insufficient quantities of ribosomal components (either RNAs or proteins) and, therefore, complete functional ribosomes cannot be formed. There may also be incorporation of viral RNA and/or viral proteins into the RNPs. Also, overexpression of X-linked spermine synthase and/or spermidine/spermine N1 acetyltransferase could result in abnormal types and levels of polyamines in the nucleolus and nucleolar fragments. For example, there may be putrescine, acetylated polyamines, and/or nuclear aggregates of polyamines in the nucleolus that interfere with RNA folding. Normally one would expect only spermine and spermidine to be present in large quantities. Extracellular release (by apoptosis, necrosis, NETosis) of these abnormal nucleolar products could provoke an autoimmune reaction that later targets the more abundant normal products due to epitope spreading.

There are over 1,000,000 Alu elements spread throughout the human genome but most are suppressed by a positioned nucleosome. Displacement of the nucleosome could open the Alu element’s internal RNA pol III transcription start site. RNA pol III can be quite prolific since it does not require energy (ATP), does not require extensive assembly of transcription factors, can initiate from the intragenic promoter in Alu elements, and can rapidly reinitiate to generate multiple transcripts. In addition, since Alu elements average only 300 bp and the intragenic promoter requires only about 70 bp for transcription factor binding, displacement of only one nucleosome would be all the opening needed. The abundance of RNA pol III typically found near the nucleolus, particularly the perinucleolar compartment, could rapidly generate thousands of Alu RNAs if there were a disruptive event, such as encroachment of the nucleolus into the Barr body. Especially vulnerable is the dense cluster of Alu elements in the PAR1 region near the surface of the Barr body. Whereas Alu elements comprise 10.8% of the human genome, they are at only 8% in the X chromosome. However, Alu elements account for 28.8% of the PAR1 region and 19% of the adjacent S5 region (46). Since PAR1 has approximately 2.5 million base pairs, there are estimated to be more than 2,500 Alu elements in PAR1 that could potentially flood the nucleus and nucleolus with Alu RNA transcripts (Figure 4A). Contrast this with the approximately 200 active ribosomal RNA genes in the nucleolus. The Alu RNAs could interfere with assembly of the SRP which contains an Alu domain that binds SRP 9/14 heterodimers (72). Free Alu RNA could compete in binding the SRP 9/14 leaving incomplete SRPs that cannot halt ribosomal activity in the cytoplasm when needed during synthesis of extracellular targeted proteins. This could lead to improper modifications (e.g., transglutamination) and localization of proteins. And, opening of the Alu elements, which have extensive intra-strand matching sequences, could also facilitate formation of cruciforms in the DNA which could be stabilized by polyamines. These cruciforms could be interpreted as autoantigens by the immune system.

Perhaps, the greatest danger from expression of Alu elements from the disrupted Barr body is deduced from the work of Caudron-Herger and colleagues mentioned previously (59). An abundance of RNA pol III Alu transcripts from the disrupted Barr body could compete with or lead to degradation of the RNA pol II intronic Alu RNA that, along with nucleolin and nucleophosmin, provides structural integrity for the nucleolus. This would lead to fragmentation of the nucleolus into inefficient subunits (Figure 4B). These nucleolar-derived subunits could have abnormal levels of polyamines and acetylated polyamines that cannot properly fold RNA and assemble RNPs. In fact, the needed components for assembly of an RNP like the ribosome may be unequally distributed among the nucleolar fragments preventing complete assembly. And there could be viral proteins and RNAs competing to join RNP assemblies. The RNPs and partial assemblies could be stabilized in abnormal conformations and associations by the polyamines and become autoantigenic when released from the cell. Such extracellular exposure could occur by blebbing and microparticle release as the cell enters apoptosis, NETosis or other forms of termination (92). With a loss of integrity of the nucleolus and expression of viral components, some nucleolar material could be displayed on the cell surface, such as the La protein (81). In addition, nucleolar fragmentation could lead to loss of nucleolar control of cell cycling, such as assembly of centromeres. Another problem that could arise is involvement of cyclic GMP-AMP synthase (cGAS) which detects cytosolic DNA. This includes detection of micronuclei that contain DNA from a disrupted nucleus or from DNA damage (93). Fragmentation of the nucleolus, as described above, could potentially generate such micronuclei, especially when centromere assembly and functioning are disrupted or when there are lagging chromosomes during mitotic segregation of chromosomes. The appearance of hypomethylated reverse transcribed Alu DNA in the cytosol, possibly originating from X-linked LINE-1 reverse transcription of PAR1 Alu element RNA, could also trigger the cGAS-STING pathway. Formation of cyclic GMP-AMP (cGAMP) can trigger activation of the Stimulator of Interferon Genes (STING) protein which induces transcription of interferon β (IFNβ) as part of the innate immune response in antiviral, antibacterial, and anticancer activity and is suspected of involvement in autoinflammatory and autoimmune diseases (94).

Therefore, the original “X chromosome–nucleolus nexus” hypothesis, which explained how the nucleolus could disrupt the inactive X chromosome, can now be expanded to include disruption of the nucleolus by X-linked Alu RNA transcripts that lead to nucleolar fragmentation and generation of autoantigenic material.

## The “X Chromosome–Nucleolus Nexus” Hypothesis in Relation to Other Diseases

The “X chromosome–nucleolus nexus” hypothesis was developed primarily with SLE in mind but it could be involved in many autoimmune diseases. The mechanism could have differing effects due to the cell types and locations involved. For example, RA and SLE can have some of the same autoantigens targeted, such as Z-DNA, but RA is primarily behind the synovial membrane reducing full exposure of antigenic and autoantigenic material to the immune system. However, continual attraction of neutrophils to the same confined inflammation site in RA where the neutrophils undergo NETosis in an ineffective attempt at clearing abnormal material would produce chronic local exposure of cells and extracellular material to the neutrophil’s active peptidyl arginine deiminases (PADs) producing high levels of citrullinated proteins (e.g., collagen) that eventually provokes the adaptive immune system into producing autoantibodies targeting the modified collagen as a major autoantigen in RA (85). In a similar manner, MS is confined behind the blood-brain barrier reducing access of autoantigenic material to the immune system but, again, neutrophils continually attracted to an MS lesion could be releasing PADs that citrullinate myelin, eventually triggering autoantibodies to citrullinated myelin basic protein which is a major autoantigen in MS. SLE is a systemic disease suggesting that the immune system can more readily react to the broad array of abnormal material seen in SLE and generate autoantibodies. Compared to RA or MS, the autoantigens, autoantibodies and complexes of the two in SLE have easier access to the circulatory system allowing the reaction to spread to and deposit in different organs rather than being confined behind a membrane barrier. SjS can be a primary disease or it can be secondary to SLE, RA or MS. This suggests that there could be similar mechanisms occurring in all four of these disorders. Involvement of polyamines, possibly due to loss of epigenetic control of X-linked polyamine genes, is suspected in SjS since the appearance of acrolein conjugated proteins is related to the intensity of SjS and acrolein is an oxidation product of polyamines (88). The hypothesis may even play a role in Alzheimer’s disease (ALZ) since there is a female bias in the disease and there are autoantibodies involved in ALZ (95). In addition, polyamine levels are altered in ALZ (96, 97) along with increased acrolein (98); SAM levels are greatly decreased (99); polyamines are involved in plaque formation (100) and nucleolar poly (ADP-ribose) polymerase 1 (PARP1) is decreased in ALZ (101). Cellular stress that leads to disruption of the inactive X chromosome and/or the nucleolus could play a role in the altered polyamine activity, decreases in SAM, decreases in PARP1, and appearance of acrolein.

The hypothesis as a whole or in parts (part 1: inactive X disruption and/or part 2: nucleolar disruption) could have a role in some cancers. The inactive X chromosome is often missing in tumor cells from breast and ovarian cancers (102). The inactive X may have reactivated from a decrease in methylation (possibly due to over activity of polyamine synthesis and recycling) or the inactive X was lost due to improper segregation of chromosomes to daughter cells (possibly due to centromere assembly in the nucleolus). Viruses could be involved in the loss of X inactivation since viruses increase polyamine levels in order to increase nucleolar activity for their benefit, as exemplified by EBV induction of ODC, SDS, and SMS via increased MYC activity. The subsequent reduction in SAM due to polyamine synthesis would make it difficult for the cell to maintain chromatin methylation required for epigenetic silencing in the X chromosome and other chromosomes leading to disruption of control of oncogenes and tumor suppressor genes and there is the possibility of opening previously sequestered viruses that then try to take control of the nucleoli. The inactive X has the greatest demands for methylation but it is the last chromosome to be replicated and repackaged in late S phase or even early G2 when SAM levels would have already been impacted by methylation of other chromosomes. Reactivation or loss of the inactive X chromosome could explain some of the cases of triple-negative breast cancer in which there is no overexpression of HER2, estrogen, or progesterone receptors (103). So this epigenetic scenario of Barr body disruption could explain some of the enigmatic cases of cancers. Also, keep in mind that viral disruption of nucleoli could interfere with nucleolar involvement in DNA repair, nucleolar assembly of centromeres, and alter nucleolar control of cell-cycle kinases (78). Fragmenting of nucleoli as centromeres are being formed could lead to abnormal distribution of chromosomes resulting in daughter cells of differing karyotypes, such as a parent (46,XX) cell generating daughter cells of (45,X0) and (47,XXX). Disruption of the nucleolus in tumor cells could also lead to appearance of autoantigens. Autoantigens can arise in cancers but they differ from those normally seen in autoimmune diseases such as SLE. The differences could arise from: the cell type involved (proliferating versus mature, differentiated); the nucleolar activity and content at the time of disruption; and the rapidity of the disruption (acute versus gradual accumulation). We can consider that nucleoli in proliferating cells would be heavily involved in cell cycling, such as generating centromere components, and so many of the autoantigens that arise would be expected to be related to cell cycling and suppression of apoptosis (104). Autoimmune diseases, such as SLE, give rise to autoantigens that are components more routinely found in abundance in nucleoli, such as ribosomal or splicosomal components.

There is a slightly higher risk of cancers among autoimmune patients but the risk varies with regards to the type of cancer (105). Therapeutics taken by the patient targeting the autoimmune disease could contribute to cancer development. Hematological, thyroid, lung, and vulva cancers show an increased risk with non-Hodgkin’s lymphoma showing a 3× to 4× greater risk in lupus patients, while breast, endometrial, and ovarian cancers show a lower risk. For now there is no direct connection between viruses and the “X chromosome–nucleolus nexus” hypothesis to the increased risk of cancers among autoimmune disease patients but we can consider the induction by viruses of increased polyamine levels and the possible reactivation of X-linked polyamine genes as means by which competition for the cellular methyl donor, SAM, could reduce DNA methylation and open oncogenes for overexpression in proliferation competent cells. Increased nucleolar size due to increased polyamines could add to the disruption of neighboring epigenetically silenced chromatin to expose alleles for expression.

## Conclusion

The focus of this discussion has been on viruses and the “X chromosome–nucleolus nexus” hypothesis since we now understand the effects a virus can have on the nucleolus and how it is to the benefit of the virus to influence the nucleolar activity. And EBV has been used as the primary example of viral involvement in autoimmune diseases since it is one of the viruses most suspected of having such a role and we can connect EBV actions (e.g., increased MYC activity) to increases in polyamines that could directly impact the nucleolus and trigger the hypothesized mechanism. Other factors besides viruses, such as bacteria or chemicals, can contribute to autoimmune diseases but the means is less clear and may not closely follow the mechanism proposed. Bacteria, for example, produce putrescine and spermidine without the extensive controls on polyamine synthesis seen in eukaryotes but the bacteria can produce their own machinery and are not as dependent as viruses on the cell’s nucleolus. In addition, disruption of heterochromatin, such as the inactive X chromosome, can open latent viruses. The X chromosome has four major fragile sites and fragile sites are frequently the locations chosen for viral insertion. The particulars of the fragile site and its vulnerability to viral insertion may add to the genetic susceptibility of an individual.

The previous version of the “X chromosome–nucleolus nexus” hypothesis, or simply the “nucleolus” hypothesis, explained how an overly stressed nucleolus could disrupt neighboring heterochromatin, especially the Barr body. As a result, there could be detrimental increases in synthesis and recycling of polyamines that could impact cellular methylation and potentially stabilize autoantigenic complexes of nucleolar and chromatin components. The point was made that many autoantigens in SLE are, at least transiently, components of the nucleolus but the means by which such nucleolar components could become autoantigenic was not presented. Now, with this work, the hypothesis has been extended to include disruption of the nucleolus as an additional step. A disrupted Barr body could generate an abundance of polyamines and Alu RNA from X-linked genes and elements that further stress and damage the nucleolus, making it very inefficient in its functions, even fragmenting it and possibly leading to cell death. And there may be overexpression of SAT1 that hampers the nucleolus in subsequent stress events since polyamines may be converted to a predominance of acetylated polyamines that are less effective at or even detrimental to proper nucleolar folding and assembly of RNAs and RNPs. Meanwhile during nucleolar disruption autoantigens may be created, stabilized and released extracellularly.

Further work is needed to understand how the various autoantigens provoke the autoimmune response. Is it, for example, a conformational alteration of the ribosomal subunits stabilized by polyamines, or incomplete assembly of an RNP? Or could it be “guilt by association” such as SSA/Ro bound to misfolded RNAs stabilized with polyamines that prevent proper refolding? There may be incorporation of viral components in the RNPs that make it autoantigenic. Or could it be abnormal localization and/or modification of proteins that are misdirected due to Alu RNA interference with SRP assembly (72). Epitope spreading from the autoantigenic complex to the normal endogenous protein would seem to have a role in the autoimmune response with the greater abundance of the endogenous protein then providing more of the provocation than the original autoantigenic complex. And the fragmentation of nucleoli, as described here, could lead to extracellular signaling and extracellular exposure of autoantigens. Testing of the hypothesis can use powerful approaches, such as computational molecular dynamics and single cell analysis, that have now reached sophistication that allow us to explore the interactions of nucleolar components as they are normally processed and the possibilities of how abnormalities could occur. For example, what is the effect of acetylated polyamines if they were to compete with spermidine and spermine in the nucleolar folding and assembly of RNPs? What are the interactions and resulting structures of intronic Alu RNA with nucleolin? And what is the distribution and composition of RNAs and proteins in nucleolar fragments compared to intact nucleoli? And, perhaps most important, what does this new hypothesis present as far as therapeutic targets? Certainly suppressing viral activity, MYC activity, polyamine synthesis, and polyamine recycling are important targets but also newer areas, such as the cGAS-STING pathway are promising targets too. The importance of Alu elements implied by this hypothesis calls into question the use of mouse models of autoimmune diseases since mice do not have the extensive amount of Alu elements seen in humans (certainly not a cluster of 28.8% seen in the PAR1 of the human X). In addition, the mouse X chromosome is telocentric (just one long arm) whereas the human X is submetacentric (a long arm and a short arm). The mouse X inactivation would be relatively consistent since it does not negotiate a centromere. X inactivation researchers complain that it is difficult to study partial X reactivation in mice due to the consistency of inactivation along the mouse X. This makes the murine X more robust under stress, at least with regards to this hypothesis (1).

We have previously discussed the short arm of the X chromosome (Xp) and especially the portion from Xp21.2 to the terminus as having a major role in SLE, particularly with regards to possible reactivation of the inactive X chromosome (1–3, 72, 82, 106, 107). This section includes: a “hot” LINE-1 (codes for a fully functional reverse transcriptase); the polyamine genes spermidine/spermine N1 acetyltransferase and SMS; and the PAR1 region with an abundance of Alu elements. Other groups are just now coming to the conclusion that the Xp arm has a major role in autoimmune diseases (108) although they have not mentioned the Alu elements, LINE-1 and polyamine genes we have mentioned and they have not made the connection to the nucleolus. It is hoped that autoimmune disease researchers will consider the “X chromosome–nucleolus nexus” hypothesis since it is the most comprehensive explanation yet for autoimmune diseases. It does involve areas with which those researchers may not be familiar, such as polyamines, X inactivation, epigenetics, and the nucleolus making it a rather complex scenario but autoimmune diseases are very complex phenomena.

## Author Contributions

The author confirms being the sole contributor of this work and approved it for publication.

## Conflict of Interest Statement

The author declares that the research was conducted in the absence of any commercial or financial relationships that could be construed as a potential conflict of interest.
